# Editorial: Cutting Edge Methodologies in Experimental Cardiovascular Research

**DOI:** 10.3389/fcvm.2020.621900

**Published:** 2020-12-14

**Authors:** Przemysław Błyszczuk, Kenneth R. Boheler, Gabriela Kania

**Affiliations:** ^1^Department of Rheumatology, Center of Experimental Rheumatology, University Hospital Zurich, Zurich, Switzerland; ^2^Department of Clinical Immunology, Jagiellonian University Medical College, Krakow, Poland; ^3^Department of Biomedical Engineering, Johns Hopkins School of Medicine, Baltimore, MD, United States

**Keywords:** cardiovacsular disease(s), methodolody, heart, cutting edge, therapy

Cardiovascular disease is a leading cause of death and a serious economic burden to patients and society. An estimated 18 million people die of cardiovascular disease (including coronary heart disease, stroke, and non-ischemic heart failure) annually (1), and despite improvements in the treatment of cardiovascular disease, hospitalizations, morbidity, and mortality continue to increase. Over the past decade, clinical data has also shown that certain modern medications may be harmful when applied to heart failure patients (2). This is an emerging challenge and has driven the development of novel drugs and new therapeutic strategies for the prevention and management of this devastating disease that are both cardiac-oriented and disease-specific. Continued advancements in preclinical models may provide evidence for innovative and effective treatment strategies, while experimental research requires the use of up-to-date and standardized methodologies.

This Research Topic on *Cutting Edge Methodologies in Experimental Cardiovascular Research* presents a collection of nine experimental and review articles covering a broad area of experimental cardiovascular research ranging from studies on the preclinical cell (Burja et al.; Stellato et al.; Woulfe et al.; Zuppinger) and animal models (Bacmeister et al.; Błyszczuk; Daskalopoulos et al.; Oppi et al.) to mathematical modeling (Faes et al.). Two articles represent typical methodological studies demonstrating novel or improved protocols. In the first, Woulfe et al. present a method for the isolation of myofibrils (a basic unit of a muscle) from rat primary cardiomyocytes. Experiments with these isolated myofibrils provide insight into muscle mechanics in the absence of non-motor proteins. This advancement enables the study of specific aspects of the function of the cardiomyocyte contractile apparatus that are not possible when using intact cells. Myofibrils are traditionally obtained from a small section of cardiac muscle and their isolation from cultured cardiomyocytes opens new opportunities for the *in vitro* manipulation of cultured cells prior to the isolation of myofibrils. In the second methodological study, Stellato et al. present a new approach to identifying and isolating murine cardiac fibroblasts using simple two-color flow cytometry. They showed that the defined combination of cell surface antigens (lineage markers Ter119, CD45, and CD31 vs. gp38) allowed the identification of collagen-producing fibroblasts in freshly isolated hearts of wild-type, non-transgenic mice (Stellato et al.). This method is breaking new ground for employing flow cytometry-based approaches to study cardiac fibroblasts in mouse models without the need for specific reporter mouse strain. In an experimental study, Burja et al. used an *in vitro* model of atherosclerosis to show the anti-inflammatory effects of olive leaf extracts in human coronary artery endothelial cells activated by serum amyloid A. The proof-of-concept provided by this early preclinical study demonstrates how promising candidates (here olive oil) may be employed for future therapeutic use. The next step in this bench-to-bedside approach is to validate any beneficial effects of these potential therapeutic agents in a living organism.

Currently, experimental animal models of human disease represent the most advanced platform for preclinical research. A review paper by Oppi et al. describes commonly used mouse models of atherosclerosis. Because atherosclerosis can be induced in several atherosclerosis-prone genetic mouse strains by different mechanisms, the paper provides a road map for researchers to choose the most appropriate model for a specific scientific question (Oppi et al.). In another review article on animal models, Błyszczuk focused on myocarditis and its consequence of dilated cardiomyopathy. This review presents the pathogenesis of myocarditis with possible clinical scenarios for the patients as well as the most commonly used experimental mouse models (Błyszczuk). This Research Topic also contains two experimental studies on animal models. In the first, Bacmeister et al. identified key methodological factors in the murine pressure-volume analysis under β-adrenergic stimulation. The authors emphasized the importance of establishing measurement protocols for both accuracy and the reproducibility of experimental data. In the second, Daskalopoulos et al. describe problems of data irreproducibility in animal models. As an example, the authors presented experimental results following treatment with a homologous peptide fragment of Wnt3a/5a UM206 in a mouse model of myocardial infarction. The authors go on to describe the inherent weaknesses associated with a single animal study due to the high number of uncontrollable variables (Daskalopoulos et al.). In laboratory practice, large scale, multicenter animal studies are often not possible for ethical and economic reasons. These data underscore the difficulty of assessing animal studies where the complexity of living organisms and the variability of housing conditions can affect outcomes.

An alternative to animal studies is offered by *in vitro* and *in silico* models. Zuppinger presents an overview of cardiovascular 3D cell culture technologies and discusses the advantages and drawbacks of various cardiac organoid cultures. The generation of human-induced pluripotent stem cell (iPSC)-derived cardiomyocytes have been proposed as models of human cardiac tissue, while advanced cell culture models coupled with human genetics are highly desirable in both experimental research and in regenerative medicine. However, these models have a number of limitations and must be considered premature as a replacement for animal studies. Although certain aspects of cardiovascular physiology and pathophysiology can be reliably mirrored by such *in vitro* models, it seems that the complete rejection of animal-based research is currently not possible. Mathematical modeling represents another option in cardiovascular research. Through the use of clinical data, Faes et al. built a mathematical model of coronary circulation to address the effects of the fractional flow reserve on the degree of coronary arterial stenosis. The successful implementation of a mathematical model to diagnostic procedures could reduce costs, save time, and minimize risk along with the social and economical burden for the patient.

Novel technologies and continued improvements to established protocols need to be shared across the scientific community to enhance experimental research and ensure that it is time- and cost- effective. The rapid development of machine learning, deep learning, and artificial intelligence may broaden the application of *in silico* approaches to address more complex scientific questions in the future. On a global scale, such advances would contribute to the faster translation of bench findings into clinical applications. This collection of Research Topic articles offers important insights into recent methodological advances in cardiovascular research that will help in the development of innovative treatment strategies.

## Author Contributions

PB, KB, and GK wrote manuscript. All authors contributed to the article and approved the submitted version.

## Conflict of Interest

The authors declare that the research was conducted in the absence of any commercial or financial relationships that could be construed as a potential conflict of interest.
